# Oxidative Stress and Antioxidant-Based Interventional Medicine in Ophthalmology

**DOI:** 10.3390/ph16081146

**Published:** 2023-08-12

**Authors:** Claudia Honisch, Umberto Rodella, Claudio Gatto, Paolo Ruzza, Jana D’Amato Tóthová

**Affiliations:** 1 Institute of Biomolecular Chemistry of CNR (ICB-CNR), Via F. Marzolo, 1, 35131 Padova, Italy; c.honisch@icb.cnr.it; 2 Research and Development, AL.CHI.MI.A. S.R.L., Viale Austria, 14, 35020 Ponte San Nicolờ, Italy; urodella@alchimiasrl.com (U.R.); cgatto@alchimiasrl.com (C.G.); 3 Fondazione Banca degli Occhi del Veneto (FBOV), Via Paccagnella, 11, 30174 Zelarino, Italy

**Keywords:** oxidative stress, reactive oxygen species, cataract, glaucoma, dry eye disease, diabetic retinopathy, macular degeneration, ocular surgery, pharmacological treatment

## Abstract

The different anatomical compartments of the eye are highly subjected to reactive oxygen species (ROS) generation due to internal factors, such as metabolic high oxygen consumption, as well as environmental factors, including UV light. An antioxidant defense system is endowed in the eye tissues to regulate ROS quantity and activity. When this homeostatic system is overwhelmed, oxidative stress occurs, causing cellular damage, chronic inflammation, and tissue degeneration. It also plays a significant role in the development and progression of various ocular diseases. Understanding the mechanisms underlying oxidative stress in ocular conditions is thus crucial for the development of effective prevention and treatment strategies. To track marketed products based on antioxidant substances as active ingredients, the databases of the European Medicines Agency and the U.S. Food and Drug Administration were consulted. Only a limited number of items were identified, which were either used as therapeutic treatment or during ocular surgery, including antioxidants, synthetical derivatives, or pro-drugs designed to enhance tissue permeation and activity. This review aims to provide an overview of the primary ocular pathologies associated with oxidative stress and of the available pharmacological interventions centered around antioxidant molecules. Such insights are essential for advancing the development of effective prevention and novel treatment approaches.

## 1. Introduction

**Scheme 1:**
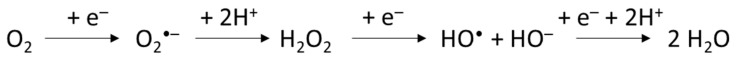
Reaction scheme for the formation of reactive oxygen species.

Oxidative stress is caused by an imbalance between the production and accumulation of reactive oxygen species (ROS) in cells and tissues and the ability of the biological system to remove these by-products. Oxygen is abundant within cells and readily accepts free electrons generated by normal metabolism producing various kinds of ROS, such as the superoxide anion radical (O_2_^•−^), hydrogen peroxide (H_2_O_2_), and the hydroxyl radical (HO^•^) (Scheme 1) [1]. These reactions are mainly localized in mitochondria in both physiological and pathological conditions [2]. Additionally, ROS may be produced by other cellular components including enzymes such as xanthine oxidase, cyclooxygenases, lipoxygenases, and the cytochrome P450-system [3]. Cellular O_2_^•−^ and H_2_O_2_ can facilitate the production of the more toxic and reactive hydroxyl radical [4], especially in the presence of reduced transition metals such as iron [5]. Moreover, the superoxide anion radical reacts with nitric oxide (NO) to form peroxynitrite (ONOO**^−^**), a strong nitrating and oxidizing molecule [6]. However, exogenous factors such as UV light, X-ray and γ-ray irradiations, air pollutants, tobacco smoke, heavy metals, and certain drugs may be involved in the generation of oxygen reactive species [7].

In physiological conditions a low intracellular concentration of ROS is maintained by the activity of the endogenous antioxidant system that comprises both enzymatic and non-enzymatic components [8]. Among the enzymatic components, we find superoxide dismutase (SOD), catalase (CAT), peroxiredoxins, glutathione peroxidases (GPXs), and glutathione reductase (GR). Non-enzymatic components are represented by a collection of molecules acting as a shield at different cellular compartments, such as ascorbic acid (vitamin C), alpha-tocopherol (vitamin E), retinol (vitamin A), molecules containing thiol groups (glutathione—GSH—and lipoic acid), and the thioredoxin system [9].

Under physiological conditions, ROS are involved as messenger molecules and in reversible post-translational modifications of key elements that exert regulatory effects on cellular functions including proliferation, differentiation, migration, and survival [10]. Protein modifications occur on both protein backbone and amino acid side chains and can be due either to ROS themselves or to secondary products of ROS-induced reactions [11].

**Figure 1:**
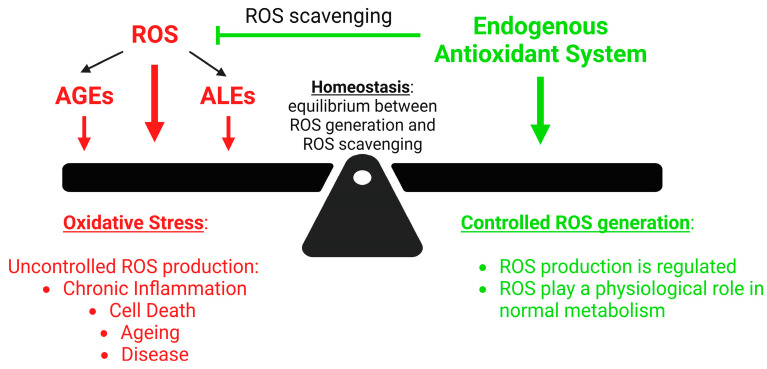
Balance between endogenous antioxidant systems and production of ROS and their by-products. Created with Biorender.com, accessed on 4th June 2023.

The insufficient activity of the endogenous antioxidant system causes the unbalanced accumulation of ROS, which leads to oxidative stress. This pro-pathological condition is characterized by a persistent high concentration of ROS that can react with proteins, lipids, and nucleic acids, in particular mitochondrial DNA [12]. The reaction of ROS with lipids of cellular membranes generates a variety of secondary cytotoxic products that in turn can react with proteins, causing covalent modifications and resulting in the generation of a family of products known as advanced lipoxidation end-products (ALEs) [13] by analogy with advanced glycation end-products (AGEs) generated by protein glycosylation [14]. Oxidative stress and oxidative-stress-induced ALE and AGE products have been implicated in aging and in a variety of age-related chronic diseases (Figure 1) [15].

The present review focuses on the role of antioxidant molecules in ocular health and diseases and on the recent advancements in antioxidant treatments developed to counteract the consequences of oxidative stress involved in the pathogenesis of several eye diseases such as senile cataract, age-related macular degeneration, uveitis, premature retinopathy, keratitis, and ocular inflammation.

## 2. Ocular Diseases Related to Oxidative Stress

The eye is a very complex organ of the human body that is exposed to several sources of oxidative stress. It is first exposed to environmental sunlight, the electromagnetic wave range of which covers the harmful UV-B and UV-C light [16], followed by the metabolic production of ROS and their by-products (ALEs, AGEs) in the aforementioned cell energy cycle [17]. Therefore, an extensive arsenal of antioxidant mechanisms is deployed in the various eye anatomical districts, including enzymatic and non-enzymatic systems, which have been reviewed in the recent publication by Rodella et al. [18]. When those antioxidant systems are overwhelmed by ROS production, depending on the site of production, different ocular diseases may arise.

### 2.1. Ocular Surface Disease

The ocular surface is almost constantly exposed to sunlight, including ultraviolet radiation, a well-recognized cause of oxidative stress [16].

Under physiological conditions, the oxidation products generated by the interaction of the radiation with tissue are faced by the activity of antioxidant enzymes, such as SOD, CAT, and GPXs. However, several factors can lead to the perturbation of this delicate balance. For instance, inflammatory cytokines such as interleukin (IL)-1, IL-6, IL-8, and tumor necrosis factor (TNF), which are up-regulated in many ocular surface diseases, increase the expression of reactive nitrogen species. When the equilibrium is displaced, a condition called Dry Eye Disease (DED) may occur.

DED affects 5% to 30% of individuals over the age of 50, distressing preferentially older people, especially women in post-menopause, contact lens wearers, and patients with autoimmune conditions [19]. Symptoms of DED include blurry vision, light sensitivity, irritation, burning, and itching, causing impairment in daily activities and having therefore a negative impact on quality of life [20].

### 2.2. Lens-Related Pathologies

#### 2.2.1. Presbyopia

Presbyopia occurs when the focusing ability of the eyes is gradually reduced with age, reaching a point where the clarity of near-sighted vision is insufficient to satisfy the individual’s requirements [21].

The focusing ability of eyes is based on accommodation, i.e., the dioptric change in lens shape in order to bring near objects into focus. The loss of accommodation capacity, among other symptoms, can also be deputed to lens hardening.

With increasing age, oxidative stress can induce changes in the viscoelastic properties of the lens. Indeed, the oxidation of protein sulfhydryl groups leads to disulphide bond formation, and such cross-linking reduces the proteins’ flexibility and results in lens stiffening [22]. The promising preliminary results of presbyopia treatment with eye drops containing antioxidant lipoic acid (see Section 3.3) further support this hypothesis.

#### 2.2.2. Cataracts

**Scheme 2:**
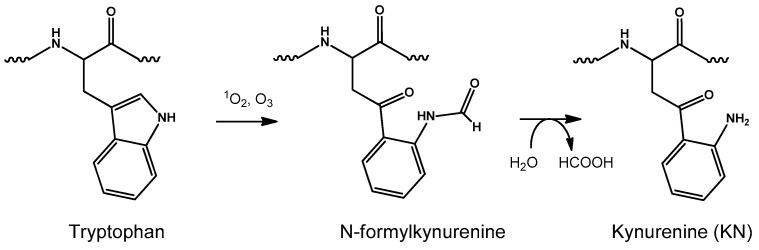
Tryptophan oxidation and its by-products.

Cataracts result from the aggregation of crystallin proteins in the eye lens, which induces lens opacification [23]. The interaction of UV radiation with crystallin aromatic residues, when exceeding their ability to dissipate energy, may damage their structure and impair the folding and stability of lens proteins, contributing to cataractogenesis. In particular, the tryptophan (Trp) indole ring may suffer oxidation and the opening of the structure, inducing a change in the hydrophobicity of the core residue, which becomes hydrophilic. Via oxidation, Trp is converted to kynurenine (Scheme 2) leading to the generation of partially unfolded conformations [24]. Oxidized Trp residues in α-, β-, and γ-crystallins have been found along with higher kynurenine content both in animal models and in human cataractous lenses compared to normal lenses of the same age [25].

Additionally, the redox homeostasis was found to be altered with aging: the synthesis and recycling mechanism of water-soluble, physiologically occurring antioxidants and radical scavengers may fail, leading to an impairment in the redox equilibrium and allowing for the formation of both intra- and intermolecular disulfide bonds in crystallins [23]. Indeed, glutathione (GSH) levels were observed to be reduced in human aged lenses and in human cataract lenses. Similarly, the levels of ascorbate were found to be lower in age-related cataractous lenses. Epidemiologic studies showed that elevated vitamin C levels are robustly related to a diminished risk of cataract development [26]. Moreover, UV radiation may interact with GSH-related lens enzymes, as observed by the UV-A and UV-B irradiation of rat pup eyes, in which a decrease in the activity of antioxidant enzymes, GR included, was observed [27]. However, despite constant exposure to oxidative stress, cataract onset is delayed throughout life by the action of both the α-crystallin chaperone activity and by the presence of physiologically occurring radical scavengers.

Besides oxidation, protein glycation, deamidation, and transglutamination also contribute to crosslinking, the subsequent insolubility of proteins, and lens opacification [28]. Moreover, the activity of antioxidant enzymes, including CAT, SOD, reductase, and enzymes of the GSH redox cycle, was found to be impaired or compromised during aging and cataract formation [28].

### 2.3. Glaucoma

Glaucoma is a chronic and progressive optic neuropathy that has a multi-factorial etiology and is characterized by a specific structural alteration of the head of the optic nerve, accompanied by progressive damage to the visual field [29]. Although its pathogenesis is not fully understood, the main histological hallmark of this condition is represented by retinal ganglion cell (RGC) death correlated with an elevated level of intra-ocular pressure. The influence of oxidative stress on the pathogenesis of glaucoma has been investigated by several authors [30,31]. Besides UV radiation, an important role is played by the oxidative stress induced by sustained endogenous aerobic metabolism, which may be supported by vascular dysregulation. Indeed, vascular alterations lead to ischemia and reperfusion, thereby inducing oxidative damage. Polyunsaturated fatty acids (PUFAs), crucial components of biological membranes, are highly sensitive to free radicals and ROS attack. When the free radicals extract an atom of hydrogen, PUFAs produce lipophilic reactive radicals and trigger lipid peroxidation, which affects both the structure and the fluidity of the cell membrane [32]. Additionally, the release of NO by the endothelium, along with the physiological tasks it exerts, may also react with superoxide anion to form peroxynitrite (ONOO**^−^**).

Oxidative stress was observed to cause the early impairment of trabecular meshwork cells, which are responsible for aqueous humor outflow and the further elevation of intra-ocular pressure [30]. Indeed, multiple reports have shown that, in the aqueous humor of patients with glaucoma, lower levels of antioxidants and elevated markers of oxidative stress could be found [33,34]. Confirming the importance of the antioxidant defense system in the prevention of glaucoma, molecules including coenzyme Q10 [35], astaxanthin [36], zeaxanthin [37], and docosahexaenoic acid [38] were shown to inhibit RGC death induced by H_2_O_2_ or by N-methyl-D-aspartate (NMDA) [39], confirming the role of oxidative stress as risk factors for RGC death [40].

### 2.4. Retinal Diseases

The retina is exposed to chronic oxidative stress by several mechanisms, including constant exposure to light and ROS generation due to high oxygen consumption by visual signal transduction pathways. Moreover, the oxidization of polyunsaturated fatty acids and the phagocytosis of photoreceptor cells occurs [41]. In a healthy state, all cell types in the retina are able to maintain homeostasis under conditions of oxidative stress. However, when the balance between pro- and anti-oxidative signaling is compromised, excessive oxidative stress induces the dysregulation of functional retinal networks and deleterious changes that result in visual impairment.

#### 2.4.1. Diabetic Retinopathy

Diabetic retinopathy (DR) is one of the most common complications of diabetes mellitus (DM) [42]. Hyperglycemia, a hallmark condition of diabetes, induces an excessive production of ROS mediated by the mitochondrial electron transport chain, which in turn leads to (i) increased polyol pathway activity with consequent sorbitol and fructose accumulation, NAD(P)H-redox imbalances, and changes in signal transduction; (ii) the non-enzymatic glycation of proteins with the production of AGEs; (iii) the activation of protein kinase C (PKC), thereby initiating a cascade of stress responses; and (iv) increased hexosamine pathway flux [43,44,45]. As a result, pathological angiogenesis is induced [46]. Such pathological vessels may cause hemorrhage or vascular leakage due to their weakness, consequently causing macular oedema, retinal ischemia, and retinal detachment.

Either the inhibition of ROS accumulation or the control of glycemic levels leads to the restoration of metabolic and vascular conditions and blocks both the initiation and progression of complications [47].

Several pre-clinical studies investigated the effect of increasing antioxidant defense by antioxidant supplementation via food administration. Several vitamins, minerals, and nutraceuticals, were identified to be useful, including lutein, zeaxanthin, vitamin C, vitamin D, vitamin E, zinc, copper, alpha-lipoic acid, N-acetylcysteine (NAC), and vitamin B complexes (B1, B2, B6, L-methylfolate, and methyl-B12) [48,49,50].

#### 2.4.2. Age-Related Macular Degeneration

Age-related macular degeneration (AMD) is a progressive degenerative disease of the retina. Early signs are represented by yellowish deposits in the retina, called drusen, that later develop in paler (hypopigmentation) or darker areas (hyperpigmentation). Advanced AMD can appear in two forms, wet and dry, both leading to visual loss. Advanced dry AMD is characterized by atrophy of the retina, leading to partial retinal detachment, while wet AMD is characterized by abnormal angiogenesis and is therefore treated with anti-VEGF drugs that inhibit the action of vascular endothelial growth factor (VEGF). Risk factors for the development of AMD include age, genetic predisposition, exposure to light, race, smoking, overweight and obesity, and diet [51].

As already mentioned, the retina shows a high metabolic rate, and it is characterized by the presence of a high concentration of PUFAs in photoreceptor cell membranes, which are prime substrates for oxidation.

In the retina there are two types of photoreceptor cells, rods and cones, which both contain the chromophore 11-cis-retinal (vitamin A1) bound to one transmembrane protein belonging to the opsins group [52]. Once light reaches photoreceptors, 11-cis-retinal is converted to its all-trans-retinal isomer, enabling the protein part (opsin) to activate the G protein transduction. All-trans-retinal is then released from the protein and reduced to all-trans-retinol and transported to the retinal pigment epithelium, where it is isomerized to 11-cis-retinol and oxidized to 11-cis-retinal, which is eventually shuttled back to the photoreceptor cell to regenerate rhodopsin [53].

The retina also contains photosensitizer molecules, chemicals which absorb light and transfer their energy to rhodopsin, including lipofuscin, melanin, and cytochrome C oxidase. In particular, lipofuscin is considered a major risk factor implicated in AMD onset and progression as it was correlated with inhibited retinal pigment epithelium (RPE) phagocytosis and photo-oxidative damage, which could induce cell death [54]. Erythrocyte-derived photosensitizers, such as protoporphyrin IX, were also described [55]. Due to its primary function, the retina is exposed to large amount of visible light, especially of shorter wavelengths, which, if coupled with inadequate levels of antioxidants, can make it very susceptible to oxidative stress, leading to lipid peroxidation in photoreceptor cell membranes and to the expression of anti-oxidative stress proteins, such as heme-oxygenase-1, causing photoreceptor cell apoptosis [56].

The Age-Related Eye Disease Study (AREDS) clinical trial, correlating antioxidant food supplementation with the onset of ocular diseases, demonstrated that oral antioxidants combined with zinc lowered the risk of progression to advanced AMD by 28% [57]. On the other hand, the subsequent AREDS2 study suggested that the addition of lutein, zeaxanthin, eicosapentaenoic acid (EPA), and docosahexaenoic acid (DHA) did not significantly reduce AMD progression compared to the original AREDS formula [58].

#### 2.4.3. Retinitis Pigmentosa and Leber Congenital Amaurosis

Retinitis pigmentosa (RP) comprises a cluster of inherited diseases represented by retinal degeneration states. If RP is not promptly treated, it may lead to blindness. Genetic mutations associated with RP are mostly related to the function and maintenance of rod photoreceptor cells [59]. Indeed, RP and Leber Congenital Amaurosis (LCA) affect the visual cycle, which refers to light being converted into an electrical signal that is ultimately conveyed to the brain [53].

In the visual cycle, the all-trans-retinal must be converted back to 11-cis-retinal via a series of enzymatic reactions. Mutations in genes involved in the visual cycle are the etiologic cause of RP and LCA diseases. Among these, the lecithin retinol acyltransferase (LRAT) and the retinoid isomerohydrolase (RPE65) enzymes are the most common. In RP and LCA the inability to produce a sufficient amount of 11-cis-retinal, as well as to remove the excess of retinoid products, leads to photoreceptor degeneration and the progressive loss of vision. As they decline in number, the metabolic consumption of oxygen is diminished, allowing for higher oxygen levels in the outer retina, which in turn leads to ROS (particularly superoxide) generation in the mitochondria and cytoplasm. The production of radicals and reactive oxygen species induces damage to DNA, lipids, and proteins, eventually leading to cone cell death [60,61].

## 3. Antioxidants in Pharmaceuticals

Given the crucial role of oxidative processes in the development of several ocular diseases, a number of studies investigated the possible applications of antioxidant molecules in ophthalmic interventional medicine, i.e., medical devices and drugs.

Drugs are subject to rigorous standards of quality, safety, and efficacy, overseen by regulatory agencies such as the Food and Drug Administration (FDA) in the United States and the European Medicines Agency (EMA) in Europe [62,63]. These medications consist of active pharmaceutical ingredients (APIs) specifically formulated to treat or prevent human diseases. Their administration aims to restore, correct, or modify physiological functions via pharmacological, metabolic, or immunological activities, as defined by Directive 2003/94/CE.

In contrast, medical devices (MDs) achieve their intended purpose without relying on pharmacological, metabolic, or immunological mechanisms. According to EMA regulations, medical devices encompass a wide range of instruments, apparatuses, appliances, software, materials, or other articles designed for diagnostic and/or therapeutic use in humans [64].

On the other hand, food supplements, as defined by both the European Food Safety Authority (EFSA) and the FDA, are products that contain concentrated amounts of nutrients or other substances intended to supplement a regular diet. While they adhere to Hazard Analysis and Critical Control Points (HACCP) regulations applicable to food products [65], they are formulated in a “pharmaceutical” format, such as pills, powders, tablets, capsules, or granules.

In order to shed light on the role of antioxidant molecules in the ophthalmic pharmaceutical field, the presence of antioxidants in the APIs of drugs intended for ophthalmic use was investigated in the EMA and FDA databases. Consultation of the abovementioned databases was performed in March 2023.

EMA drugs database (https://www.ema.europa.eu/en/medicines, accessed on 1 March 2023) gives the opportunity to group drugs by therapeutic area. Exploiting this option, the following therapeutic areas were selected, obtaining a total of *n* = 89 non-redundant reference drugs entries: *-conjunctivitis*; *-conjunctival disease*; *-conjunctivitis allergic*; *-corneal diseases*; *-diabetic retinopathy*; *-dry eye syndrome*; *-eye discomfort*; *-eye diseases*; *-eye diseases (hereditary)*; *-glaucoma*; *-glaucoma (open-angle)*; *-myopia; -myopia (degenerative)*; *-neuromyelitis optica*; *-ocular hypertension*; *-ophthalmology*; *-optic atrophies (hereditary)*; *-optic atrophy*; *-optic atrophy (Hereditary, Leber)*; *-optic nerve diseases*; *-optic neuritis*; *-retinal degeneration*; *-retinal diseases*; *-retinal dystrophies*; *-retinitis pigmentosa*.

The 89 entries were then scanned for antioxidant molecule(s) in the drugs’ ingredients, eventually obtaining *n* = 4 entries (see Table 1), three of which are not currently on the market and are part of a Pediatric Evaluation Plan (PIP).

Differently from the EMA database, although it does not provide the option to categorize drugs based on therapeutic areas, the FDA database (https://www.fda.gov/drugs/drug-approvals-and-databases/drugsfda-data-files, accessed on 1 March 2023) permits users to download a dataset (Drugs@FDA Download File ZIP), which allows them to index drugs (different formats and generic drugs included) based on certain characteristics, such as the API.

Drugs containing antioxidants as active ingredients were initially filtered starting from the complete FDA dataset. Hence, in a second step, the latter have been further filtered by selecting drugs employed in ophthalmology, obtaining *n* = 6 entries (see Table 1). In the following paragraphs, the identified pharmaceuticals are discussed in more detail. In addition, an overview of oxidative-stress-related ophthalmic pathologies as well as the antioxidant-based strategies in interventional medicines are summarized in Table 2.

### 3.1. Cysteamine in the Treatment of Ophthalmic Cystinosis

**Figure 2:**
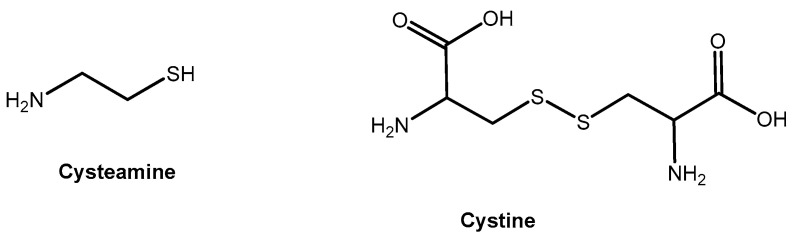
Chemical structure of cysteamine and cystine, the oxidized dimer of the amino acid cysteine.

Cysteamine (Figure 2), also known as mercaptamine, is a small aminothiol. It is the active substance in drugs employed in the treatment of cystinosis, a rare and autosomal recessive disorder caused by a mutation in the gene *CTNS*, which codes for cystinosis, the lysosomal cystine transporter [67]. In cystinosis, the lysosomal transport of cystine, the oxidized dimer form of the amino acid cysteine, is defective, causing cystine crystal accumulation in various body tissues, including the cornea, mostly in the stroma and in the peripheral layer [66].

The eye drops formulation Cystaran^®^ (cysteamine hydrochloride ophthalmic solution 0.44%, Leadiant Biosciences Inc., Rockville, MD, USA) received FDA approval in 2012 for the treatment of cystine crystal corneal deposits in cystinosis. Differently, an analogous drug, Dropcys (cysteamine hydrochloride ophthalmic solution 0.1%, Lucane Pharma, Paris, France) was refused by the EMA in 2015. On the other hand, Cystadrops^®^ (cysteamine hydrochloride ophthalmic gel 0.37%, Recordati Rare Diseases Inc., Lebanon, NJ, USA) is an ocular topical gel authorized both by the EMA and FDA for the treatment of eye crystal deposits in cystinosis.

When applied topically as eye drops or gel, cysteamine enters into lysosomes of corneal cells and reduces cystine disulfide bonds so that the products of the redox reaction can exit from lysosomes, abating the formation of crystals [67].

None of the clinical studies on cysteamine-based drugs reported any serious adverse event in patients. However, the use of cysteamine-based medicines exhibits some limitations. Firstly, the hydrophilic cysteamine is characterized by poor penetration through the lipophilic corneal epithelium, even if it was found that complexation with alpha-cyclodextrins and neutral pH allows greater penetrance through the corneal layers [74]. Secondly, cysteamine is unstable in aqueous solution at room temperature, oxidizing to the inactive disulphide dimer (Figure 2). In addition, cysteamine has a brief residence time on the cornea, thus requiring frequent administration (up to 12 times per day), with likely poor compliance by the patients [67].

### 3.2. Zuretinol as a Potential Oral Treatment for Retinitis Pigmentosa and Leber Congenital Amaurosis

**Figure 3:**
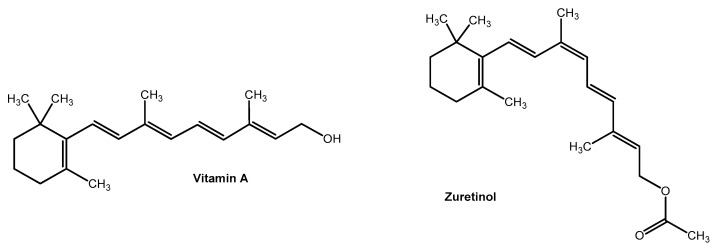
Vitamin A (retinol) and the synthetic derivative zuretinol.

Vitamin A (Figure 3) belongs to the class of carotenoids and plays a crucial role in the sight process by interaction with the photoreceptors opsin and rhodopsin in the form of 11-cis-retinal [75]. The antioxidant activity of vitamin A is mediated by its metabolite all-trans-retinoic acid (ATRA), which was observed to regulate expression levels of a battery of target genes involved in responses to oxidative stress [76].

Zuretinol (9-cis-retinyl-acetate; Figure 3) is a synthetic derivative of the fat-soluble vitamin A that completed a clinical trial (ClinicalTrials.gov identifier NCT01014052) as oral medicine (QLT091001) for the treatment of Leber Congenital Amaurosis (LCA) or retinitis pigmentosa (RP) in 2013. Moreover, the EMA agreed to a pediatric investigation plan (EMEA-001453-PIP01-13-M01) for the evaluation of safety and efficacy in pediatric populations. In this context, zuretinol (QLT091001) has shown to be promising in the treatment of RP and LCA caused by mutations in the LRAT and RPE65 genes as it combines with opsin to form isorhodopsin, which is also capable of starting the photo-transduction cascade when activated by light [77].

In models of LRAT and RPE65 double knock-out mice, oral treatment with zuretinol prevented the loss of cone photoreceptors [78]. Notably, in an open-label, prospective, phase 1b clinical trial (NCT01014052), 14 LCA patients (aged 6–38 years) received seven days of oral QLT091001 (10–40 mg/m^2^/day). After two years, 10 patients had an improvement in Goldmann visual fields areas, 6 had improved visual acuity, and no serious adverse events were reported [77,79]. These initial findings suggest that zuretinol holds promise as a potential treatment for LCA patients. However, further studies are necessary to validate its efficacy given the limited sample size of the human study. Additionally, a limitation of this treatment is the requirement for the continuous oral administration of the drug, which may pose challenges to patient compliance.

### 3.3. Lipoic Acid Choline Ester in the Treatment of Presbyopia

**Figure 4:**
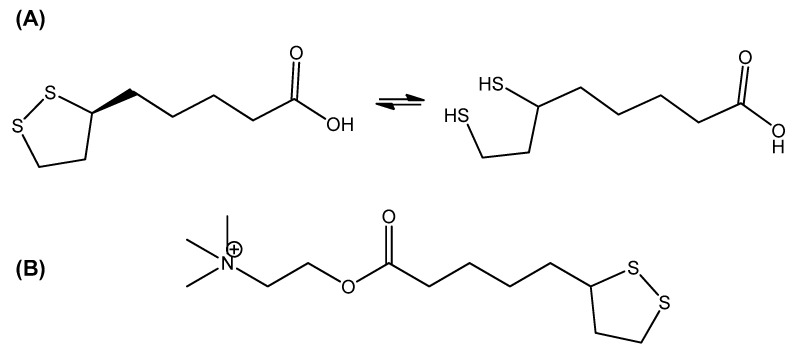
(**A**) Alpha-lipoic acid is a cyclic disulfur that is in equilibrium with the linear reduced form. (**B**) Dihydrolipoic acid α-lipoic acid choline ester.

α-lipoic acid (LA) is a naturally occurring organosulfur with antioxidant and anti-inflammatory properties. It shows direct antioxidant activity via redox chemistry of the disulfuric moiety (Figure 4A) and is also involved in the regeneration of other antioxidant molecules, such as GSH, vitamin C, and vitamin E [68].

An α-lipoic-acid-based drug is currently under study for the pharmacological treatment of presbyopia. UNR844 (formerly known as EV06, Novartis Europharm Ltd., Horsham, UK) is an eye drops formulation of α-lipoic acid choline ester (LACE, Figure 4B) 1.5% and has completed a prospective, randomized, double-masked, placebo-controlled multicenter Phase I/II exploratory study [80]. UNR844 is also under evaluation by the European Medicine Agency (EMA) for pediatric use (Pediatric Investigation Plan EMEA-002811-PIP01-20).

UNR844 is designed to be a pro-drug, for which the active ingredient is LA. Preliminary studies showed that the incubation of isolated mouse lenses with LA resulted in a dose-dependent reduction in disulfide bonds in crystallin proteins and in higher flexibility of the lens [22]. However, LA does not penetrate the cornea in vivo. On the other hand, LACE permeates corneal layers thanks to its higher hydrophobicity, and it is metabolized into choline and LA by corneal esterases. It was demonstrated that the LA concentration in aqueous humor rises following topical ocular treatment with LACE [22]. Thereby, oxidoreductases within lens cells chemically reduce LA to the active form, dihydrolipoic acid (DHLA), which exerts its antioxidant activity [81]. Indeed, the topical application of LACE eye drops reduces disulfide bonds between lens proteins and increases lens elasticity, as shown in an in vivo murine model [22].

The safety and efficacy of UNR844 (LACE 1.5% eye drops) was assessed in human subjects with presbyopia in a Phase I/II exploratory study (ClinicalTrials.gov identifier NCT02516306) [80]. The study evaluated the ophthalmic solution safety when applied topically and its efficacy in improving distance-corrected near visual acuity (DCNVA) in subjects with presbyopia. The study observed a statistically significant higher improvement of DCNVA in the group receiving UNR844 (*n* = 50) twice a day for 91 days, in comparison to the placebo group (*n* = 25), both in the treated eye as well as in bilateral vision. The DCNVA was significantly higher in the UNR844 group (*n* = 34) than in the placebo group (*n* = 18) also at 5- and 7-month follow-up after the final dosing with UNR844. The low number of adverse events suggested that UNR844 is well tolerated and raises no safety concerns, further supporting the development of LACE ophthalmic solution for the treatment of presbyopia [80].

### 3.4. DHA in the Treatment of Retinitis Pigmentosa

Omega-3 fatty acids exert their beneficial activity by regulating second messengers’ systems. In particular, in vitro studies suggested their role in increased cellular levels of GSH as well as enzymatic antioxidants such as SOD and GPXs [82,83]. Among them, DHA is a long chain ω-3 PUFA particularly enriched in some fish species (i.e., salmon, mackerel, and anchovies), which is essential for retinal photoreceptor integrity and for the photo-transduction cascade [84]. It also counteracts lipid peroxidation that may occur at the cellular membranes [85].

Two clinical trials were performed and completed in 2015 in order to verify the safety and efficacy of oral DHA in the treatment of X-linked RP (ClinicalTrials.gov Identifiers: NCT00100230 and NCT00004827). The publications arising from these trials come to, at least partly, conflicting conclusions.

In the safety assessment of an oral dose of 30 mg/kg/day of DHA, red blood cell DHA levels were increased by 3.6 times after 4 years of treatment, and the absence of serious adverse events indicated limited safety risks in long-term oral DHA intake [86].

Hoffmann and colleagues reported in 2014 the results of a 4-year, randomized, double-masked, phase 2 clinical trial where 78 X-linked RP patients were assigned to oral algal-derived DHA (30 mg/kg/day, *n* = 33) treatment or a placebo (*n* = 27) [87]. The electroretinography analyses indicated that long-term oral DHA administration was not effective in slowing the loss of cone or rod function associated with X-linked RP. On the other hand, the same group observed in a later publication that oral DHA treatment reduced the rate of progression of X-linked RP in final dark-adapted thresholds and visual field sensitivity [88].

Based also on non-conclusive clinical results, in 2021, the EMA refused a Pediatric Investigation Plan (EMEA-002811-PIP01-20) for the treatment of RP with oral DHA, “on the grounds that the specific medicinal product is likely to be ineffective or unsafe in part or all of the pediatric population”. It is important to highlight that besides being used as a drug for RP patients, DHA is also a nutrient naturally present in various foods in the human diet. As noted in other reviews [18], demonstrating the pharmacological effect of dietary molecules can be more challenging compared to artificially synthesized drugs. Despite the rejection by the EMA as a drug for RP, DHA and other PUFAs may still have a potential role in alleviating symptoms when incorporated into the diet.

## 4. Antioxidants in Ocular Surgery

Eye surgery includes refractive and vitreoretinal surgery corresponding to the anterior and posterior segments of the eye, respectively. Refractive surgery corrects or minimizes refractive errors and is based on two main surgical approaches: keratorefractive surgery, which alters corneal surface shape, and intraocular lens (IOL)-based surgery, in which an IOL implant is added to the optical elements. Among vitreoretinal surgeries, pars plana vitrectomy (PPV) involves the removal of the humor vitreous from the posterior chamber before accessing the diseased part of the retina for treating conditions like retinal holes, retinal detachment, or for removing retinal pathological membranes. PPV represents the third most common intraocular procedure performed in the United States [89].

The abovementioned interventions normally require a varying degree of invasiveness as well as the use of intraocular illumination probes to visualize the tissues to be manipulated. Given this, it is possible that the mechanical and light stresses that ensue may result in the direct and/or indirect production of radicals in the ocular tissues. In particular, photochemical toxicity occurs when the ocular tissues are exposed to UV and shorter wavelength radiation of light [90]. During surgery, light sources can also cause thermal damage to eye tissues, especially due to their proximity [91]. Moreover, in some interventions, the endogenous antioxidant system of the eye is partly removed as in PPV and IOL-based refractive surgery [92,93]. Accordingly, an increased incidence in cataracts or glaucoma was observed in patients following vitrectomy due to antioxidant depletion derived from vitreous removal [94].

To prevent or mitigate the abovementioned disruption of ROS homeostasis, some medical devices and drugs containing antioxidants have been developed for use during surgery.

### 4.1. Irrigation Solutions

Irrigation solutions are sterile cleansing solutions employed during various eye surgeries with the aim of preserving as much as possible the physiology of eye tissues throughout a surgical procedure. One of the first, most known, and most employed irrigation solution is a balanced salt solution (BSS).

BSS supplemented with glutathione, a natural occurring tripeptide composed of glutamine, cysteine, and glycine with the ability to directly scavenge free radicals and reactive oxygen species [70], was developed in the 1970s as it proved to restore corneal thickness in a rabbit model of cold-swollen corneas. The authors found that oxidized glutathione was equally effective as reduced glutathione and the process was oxygen-dependent [95].

BSS plus^®^ (balanced salt solution enriched with bicarbonate, dextrose, and oxidized glutathione 0.184 mg/mL, Alcon Pharmaceuticals Ltd., Fribourg, Switzerland) was approved by the FDA in 1981 for irrigation during ophthalmic surgery. Successively, a new preparation for the same application and with the same content of oxidized glutathione was approved by the FDA in 2008 (Navstel, Alcon Pharmaceuticvals Ltd., Fribourg, Switzerland).

In 1981, Benson and colleagues employed BSS plus^®^ in a two-center study on 44 human DR patients undergoing PPV. The study showed that BBS plus^®^ was superior to lactated Ringer’s Solution in mitigating corneal swelling in the first postoperative day. The authors suggested that the observed difference could be attributed to the presence of GSH, which potentially contributes to the better maintenance of endothelium viability [96]. On the other hand, Rosenfeld and colleagues examined the effects of BSS plus^®^ in comparison to BSS^®^ (not containing glutathione) in 71 patients undergoing PPV and followed for at least 6 months. The study concluded that both the irrigating solutions were equally effective in preserving the integrity of the corneal endothelium, analyzed by a contact-type specular microscope [97].

Nuyts et al. compared BSS plus^®^ with other irrigating solutions in perfused ex vivo human corneas [98]. BSS plus^®^ was superior to Hartmann’s Lactated Ringer’s (HLR) solution in maintaining corneal thickness. Moreover, endothelial morphology examined by transmission and scanning electron microscopy was better preserved in BSS plus^®^-perfused corneas than in BSS^®^- or HLR-perfused corneas.

More recently, it was suggested to use BSS plus^®^ in other ophthalmic surgical techniques as well. Cameron and colleagues found that the phacoemulsification cataract surgery generates the hydroxyl radical (but not other radicals) and that its concentration is reduced in the presence of BSS plus^®^ [99].

### 4.2. Lutein

Lutein belongs to a subclass of carotenoids containing oxygen named xanthophylls [100]. They are present in the macula lutea in a concentration gradient that is highest at the center of the fovea. The role of ocular carotenoids is to absorb light, particularly blue light, which is characterized by a short wavelength and high energy, and protect eye tissues from light-induced photochemical damage. Additionally, they have the ability to quench free radicals by accepting their unpaired electrons, stabilizing them in their conjugated double-bond system [101].

An innovative and recent application of lutein is its use as a dye in PPV where its yellow-red color is exploited to improve the visualization of humor vitreous and retina membranes [102]. In parallel, a novel xanthophyll-based green dye was developed in 2014 for PPV involving retinal internal limiting membrane (ILM) and epiretinal membrane (ERM) removal [103], created by combining lutein/zeaxanthin 0.3% yellow crystals and brilliant blue 0.025%. It was observed to be safe and of easy manipulation during surgery, suggesting that the exogenous xanthophylls may be natural absorbers of the blue light introduced by intraocular illumination probes, therefore decreasing iatrogenic oxidative stress on the retina.

Further studies investigated this potential extra benefit of xanthophyll-based dyes. Despite involving few participants, two preliminary studies on human subjects seem to confirm this hypothesis. In the first study, the addition of 2% lutein to trypan blue + brilliant blue dye significantly reduced iatrogenic mechanical damage to Müller cells after PPV. Furthermore, patients recovered faster visual acuity and macular sensitivity in comparison to control groups who underwent PPV employing the blue dye devoid of lutein [104], suggesting a protective activity towards retinal photo-toxicity induced by the invasiveness and use of light probes. In the second study, a lutein + zeaxanthin dye offered the PPV intraoperative protection of photoreceptors and was significantly better than brilliant blue dye.

### 4.3. Riboflavin

Despite not being traditionally considered an antioxidant, riboflavin (vitamin B2) can be regarded as such since a number of studies suggest that, even if indirectly, riboflavin supports the endogenous antioxidant system [105].

Indeed, studies conducted on riboflavin-deficient animal models showed decreased levels of GSH and of antioxidant enzymes in different eye compartments, along with higher levels of oxidative stress markers. Moreover, riboflavin administration reversed the situation. Additionally, riboflavin was shown to enhance the antioxidant effects of vitamins C and E by contributing to the GSH redox cycle [105].

Riboflavin displays an orange-yellow color, and this feature is exploited for staining specific ocular tissues in vitreoretinal surgery and cataract removal.

Moreover, the marked ability of riboflavin to absorb UV light is employed also in corneal collagen cross-linking (CXL), a widely used surgical treatment for keratoconus patients. In the first step of such treatment, riboflavin 0.1% is applied topically on the cornea surface. Rapidly, riboflavin is diffusely distributed and reaches the aqueous humor, with a concentration inversely dependent to corneal depth [106]. In the second step, a UV-A laser beam is used to excite riboflavin. The high amount of energy discharged to riboflavin overwhelms the molecule’s absorption capacity, causing riboflavin itself to act as a photosensitizer, generating ROS in the sub-epithelial and anterior-mid stroma. As a result, the corneal collagen’s ability to form intermolecular bonds is enhanced, leading to the cross-linking of the collagen fibers in the corneal stroma. This process stabilizes the corneal structure and helps halt the progression of keratoconus.

In the context of corneal ectasia and keratoconus, Photrexa^®^ Viscous and/or Photrexa^®^ drugs are employed for the CXL process. These products contain riboflavin in a viscous solution, which enhances the absorption and distribution of riboflavin within the cornea [107].

## 5. Conclusions and Future Perspectives

The eye is a marvel of biological engineering, enabling us to perceive and comprehend the world around us. It is composed of several anatomical compartments, including the cornea, iris, lens, and retina. When light enters the eye, it first passes through the cornea and then travels through the lens, precisely focusing onto the retina. Within the retina, the light is converted into electrical signals, which are transmitted to the brain through the optic nerve.

However, the eye’s close proximity to the external environment, as well as its extremely high oxygen consumption (especially in the retina) make it vulnerable to oxidative stress. Oxidative stress refers to an imbalance between free radicals and ROS production and the body’s natural defense mechanisms, resulting in cellular damage, chronic inflammation, aging, and pro-pathological conditions. In addition, the eye is constantly exposed to light rays, including UV radiation, which can generate free radicals and exacerbate oxidative stress. The latter is a significant contributor to the development of various ocular pathologies, highlighting the importance of addressing it in ophthalmological interventional medicine.

The primary strategy in fighting ocular diseases is prevention. Protective eyewear, such as sunglasses or safety glasses, can help reduce the amount of radiation reaching the eyes, thereby minimizing oxidative stress. However, an equally crucial aspect is providing adequate nutritional support to the body’s endogenous antioxidant system. Scientific studies, including a recent review by Rodella et al. [18], have emphasized the significance of the supply of antioxidant nutrients via a balanced diet in order to support ocular health. Many of these antioxidant nutrients are derived from plants; therefore, botanical extracts hold significant potential for applications in the field of ophthalmology. However, it is important to acknowledge that the standardization of botanical extracts poses considerable challenges. Ensuring consistent quality, potency, and purity of these extracts is complex and requires rigorous testing and validation. Due to these challenges, botanical extracts have not yet gained widespread access in interventional medicine applications. When preventive measures based on nutrition and protective eyewear are insufficient, interventional medicine becomes necessary. This approach involves the use of authorized drugs, approved by regulatory agencies such as the European Medicines Agency (EMA) and the U.S. Food and Drug Administration (FDA), as well as specific medical devices. Both drugs and medical devices play crucial roles in managing ocular conditions, including their application in surgical procedures.

Drugs can be administered via various routes, including oral ingestion or topical application via eye drops. Medical devices, on the other hand, can be utilized as eye drops or during ocular surgeries.

One important advantage of drugs and medical devices containing antioxidant substances, as those described here, lies in their composition. These substances are either naturally occurring molecules or modified versions of them, such as the choline ester of lipoic acid. Their structural similarity to compounds already evolutionary familiar to our bodies allows for efficient recognition and metabolism.

Nevertheless, one limitation associated with these treatments is the challenge of demonstrating their efficacy. An additional challenge is to establish adequate formulations and routes of administration that ensure proper absorption and targeted action within the eye. Overcoming the barriers posed by the hydrophobic corneal epithelium, along with the constant flow of tears that covers the ocular surface, represents a challenge for the topical administration of active pharmaceutical ingredients into the eye. On the other hand, the oral route of administration faces numerous barriers on its path from ingestion to reaching the eye as the site of action, including the blood–ocular barrier.

In conclusion, maintaining optimal eye health requires a comprehensive approach. In the field of interventional medicine, the utilization of active ingredients with antioxidant properties is closely associated with the prevention and treatment of ocular diseases induced by oxidative stress.

## Figures and Tables

**Table 1:** Results of pharmaceutical EMA and FDA databases queries.

Database	ID	Form	Drug Name	Active Antioxidant Ingredient(s) Declared
EMA	EU designation number: EU/3/08/578	Topical application eye drops	Cystadrops	Cysteamine HCl
EMA	PIP number: EMEA-001453-PIP01-13-M01	Oral use	\	Zuretinol acetate
EMA	PIP number: EMEA-002811-PIP01-20	Topical application eye drops	\	α-R-lipoic acid choline ester
EMA	PIP number: EMEA-002808-PIP01-20	Oral use	\	Docosahexaenoic acid (DHA)
FDA	ApplNo: 18469	Solution for irrigation	BSS PLUS	CaCl_2_; desxtrose; gluthatione disulphide; MgCl_2_; KCl; NaHCO₃; NaCl; Na₃PO₄
FDA	ApplNo: 211302	Topical application eye drops	Cystadrops	Cysteamine HCl
FDA	ApplNo: 200740	Topical application eye drops	Cystaran	Cysteamine HCl
FDA	ApplNo: 22193	Solution for irrigation	Navstel	CaCl_2_; dextrose; MgCl_2_; oxiglutathione; KCl; NaHCO₃; NaCl; Na₃PO₄
FDA	ApplNo: 203324	Topical application eye drops: photoenhancer indicated for use in corneal collagen cross-linking	Photrexa	riboflavin 5′-phosphate sodium

**Table 2:** Overview of oxidative-stress-related eye pathologies and antioxidant-based approaches in ophthalmic interventional medicines.

Oxidative-Stress-Related Eye Condition		Oxidative Stress Proposed Mechanism	Antioxidant Ingredients, Drug Formulation, and Mechanism of Action
**Ocular surface disease**	**Dry Eye Disease (DED)**	Exposure to highly energetic sunlight wavelengths: ROS production and inflammation in the cornea layers [20].	Antioxidant-based interventional medicine not available to date (for prevention please refer to [18]).
	**Cystinosis**	Inherited defective metabolism of cystine, which is the oxidized dimer of cysteine, leads to cystine crystal accumulation in the cornea [66].	**- Cysteamine (hydrochloride salt)**: eye drops or gel → reduction of cystine disulphide bond [67].
**Lens-related pathologies**	**Presbyopia**	Alteration of viscoelastic properties and increased stiffness of the lens due to oxidative stress exposure [22].	**- α-Lipoic Acid Choline Ester**: eye drops, pro-drug able to cross the cornea layers → reduction of lens proteins’ disulfide bonds and increase in lens elasticity [68].
	**Cataract**	Oxidation and unfolding of lens crystallins proteins [69].	**- GSH**: irrigation solutions for IOL-based surgeries → direct scavenger of hydroxyl radical and superoxide [70].
**Glaucoma**		Oxidative stress contributes to early deterioration of trabecular meshwork [32].	Antioxidant-based interventional medicine not available to date (for prevention please refer to [18]).
**Retinal diseases**	**Diabetic retinopathy (DR)**	Hyperglycemia-induced ROS production induces pathological angiogenesis by different mechanisms, causing retinal ischemia and detachment [71].	Antioxidant-based interventional medicine not available to date (for prevention please refer to [18]).
	**Age-related macular degeneration (AMD)**	Oxidative stress + UV light + low levels of retinal antioxidants contribute to progressive retina degeneration [72].	Antioxidant-based interventional medicine not available to date (for prevention please refer to [18]).
	**Retinitis pigmentosa (RP) and Leber Congenital Amaurosis (LCA)**	Inherited induced photoreceptors degeneration causes reduced oxygen consumption in the retina, leading to higher oxygen availability in the outer retina, with consequent ROS production and cone cell death.	**- Zuretinol Acetate**: Via oral administration, it combines with opsin to form isorhodopsin, starting the redox reactions of the photo-transduction cascade → attenuation of photoreceptors degeneration. **- DHA**: oral administration → boosting of retinal antioxidant endogenous arsenal.
**Iatrogenic oxidative stress**	**Oxidative stress induced by refractive and vitreoretinal surgery**	- ROS production induced by surgical manipulation and light probes. - Surgical removal of eye tissues naturally endowed with antioxidants.	**- GSH**: irrigation solutions for eye surgeries → scavenger of free radicals and ROS [70]. **- Xantophylls (Lutein and Zeaxanthin)**: dyes in PPV → absorb blue light and interact with oxidized primary scavengers, regenerating their antioxidant ability [73].

## Data Availability

Data is contained within the article.

## References

[1] Krumova K., Gonzalo C. (2016). Overview of Reactive Oxygen Species. Singlet Oxygen: Applications in Biosciences and Nanosciences.

[2] Bruni F. (2021). Mitochondria: From Physiology to Pathology. Life.

[3] Di Meo S., Reed T.T., Venditti P., Victor V.M. (2016). Role of ROS and RNS Sources in Physiological and Pathological Conditions. Oxidative Med. Cell. Longev..

[4] Lipinski B. (2011). Hydroxyl radical and its scavengers in health and disease. Oxidative Med. Cell. Longev..

[5] Stohs S.J., Bagchi D. (1995). Oxidative mechanisms in the toxicity of metal ions. Free Radic. Biol. Med..

[6] Radi R. (2018). Oxygen radicals, nitric oxide, and peroxynitrite: Redox pathways in molecular medicine. Proc. Natl. Acad. Sci. USA.

[7] Phaniendra A., Jestadi D.B., Periyasamy L. (2015). Free radicals: Properties, sources, targets, and their implication in various diseases. Indian J. Clin. Biochem. IJCB.

[8] Ginter E., Simko V., Panakova V. (2014). Antioxidants in health and disease. Bratisl. Lek. Listy.

[9] Birben E., Sahiner U.M., Sackesen C., Erzurum S., Kalayci O. (2012). Oxidative stress and antioxidant defense. World Allergy Organ. J..

[10] Sinenko S.A., Starkova T.Y., Kuzmin A.A., Tomilin A.N. (2021). Physiological Signaling Functions of Reactive Oxygen Species in Stem Cells: From Flies to Man. Front. Cell Dev. Biol..

[11] Juan C.A., Pérez de la Lastra J.M., Plou F.J., Pérez-Lebeña E. (2021). The Chemistry of Reactive Oxygen Species (ROS) Revisited: Outlining Their Role in Biological Macromolecules (DNA, Lipids and Proteins) and Induced Pathologies. Int. J. Mol. Sci..

[12] Chao M.R., Evans M.D., Hu C.W., Ji Y., Møller P., Rossner P., Cooke M.S. (2021). Biomarkers of nucleic acid oxidation—A summary state-of-the-art. Redox Biol..

[13] Vistoli G., De Maddis D., Cipak A., Zarkovic N., Carini M., Aldini G. (2013). Advanced glycoxidation and lipoxidation end products (AGEs and ALEs): An overview of their mechanisms of formation. Free Radic. Res..

[14] Sharma C., Kaur A., Thind S.S., Singh B., Raina S. (2015). Advanced glycation End-products (AGEs): An emerging concern for processed food industries. J. Food Sci. Technol..

[15] Moldogazieva N.T., Mokhosoev I.M., Mel’nikova T.I., Porozov Y.B., Terentiev A.A. (2019). Oxidative Stress and Advanced Lipoxidation and Glycation End Products (ALEs and AGEs) in Aging and Age-Related Diseases. Oxidative Med. Cell. Longev..

[16] de Jager T.L., Cockrell A.E., Du Plessis S.S. (2017). Ultraviolet Light Induced Generation of Reactive Oxygen Species. Adv. Exp. Med. Biol..

[17] Yang S., Lian G. (2020). ROS and diseases: Role in metabolism and energy supply. Mol. Cell. Biochem..

[18] Rodella U., Honisch C., Gatto C., Ruzza P., D’Amato Tóthová J. (2023). Antioxidant Nutraceutical Strategies in the Prevention of Oxidative Stress Related Eye Diseases. Nutrients.

[19] Stapleton F., Alves M., Bunya V.Y., Jalbert I., Lekhanont K., Malet F., Na K.S., Schaumberg D., Uchino M., Vehof J. (2017). TFOS DEWS II Epidemiology Report. Ocul. Surf..

[20] Miljanović B., Dana R., Sullivan D.A., Schaumberg D.A. (2007). Impact of dry eye syndrome on vision-related quality of life. Am. J. Ophthalmol..

[21] Berdahl J., Bala C., Dhariwal M., Lemp-Hull J., Thakker D., Jawla S. (2020). Patient and Economic Burden of Presbyopia: A Systematic Literature Review. Clin. Ophthalmol. (Auckl. N.Z.).

[22] Garner W.H., Garner M.H. (2016). Protein Disulfide Levels and Lens Elasticity Modulation: Applications for Presbyopia. Investig. Ophthalmol. Vis. Sci..

[23] Serebryany E., Takata T., Erickson E., Schafheimer N., Wang Y., King J.A. (2016). Aggregation of Trp > Glu point mutants of human gamma-D crystallin provides a model for hereditary or UV-induced cataract. Protein Sci. A Publ. Protein Soc..

[24] Xia Z., Yang Z., Huynh T., King J.A., Zhou R. (2013). UV-radiation Induced Disruption of Dry-Cavities in Human γD-crystallin Results in Decreased Stability and Faster Unfolding. Sci. Rep..

[25] Zhang J., Yan H., Löfgren S., Tian X., Lou M.F. (2012). Ultraviolet radiation-induced cataract in mice: The effect of age and the potential biochemical mechanism. Investig. Ophthalmol. Vis. Sci..

[26] Yonova-Doing E., Forkin Z.A., Hysi P.G., Williams K.M., Spector T.D., Gilbert C.E., Hammond C.J. (2016). Genetic and Dietary Factors Influencing the Progression of Nuclear Cataract. Ophthalmology.

[27] Fan X., Zhou S., Wang B., Hom G., Guo M., Li B., Yang J., Vaysburg D., Monnier V.M. (2015). Evidence of Highly Conserved β-Crystallin Disulfidome that Can be Mimicked by In Vitro Oxidation in Age-related Human Cataract and Glutathione Depleted Mouse Lens. Mol. Cell. Proteom..

[28] Sharma K.K., Santhoshkumar P. (2009). Lens aging: Effects of crystallins. Biochim. Biophys. Acta.

[29] Rolle T., Ponzetto A., Malinverni L. (2020). The Role of Neuroinflammation in Glaucoma: An Update on Molecular Mechanisms and New Therapeutic Options. Front. Neurol..

[30] Fernández-Durango R., Fernández-Martínez A., García-Feijoo J., Castillo A., de la Casa J.M., García-Bueno B., Pérez-Nievas B.G., Fernández-Cruz A., Leza J.C. (2008). Expression of nitrotyrosine and oxidative consequences in the trabecular meshwork of patients with primary open-angle glaucoma. Investig. Ophthalmol. Vis. Sci..

[31] Zanon-Moreno V., Marco-Ventura P., Lleo-Perez A., Pons-Vazquez S., Garcia-Medina J.J., Vinuesa-Silva I., Moreno-Nadal M.A., Pinazo-Duran M.D. (2008). Oxidative stress in primary open-angle glaucoma. J. Glaucoma.

[32] Izzotti A., Bagnis A., Saccà S.C. (2006). The role of oxidative stress in glaucoma. Mutat. Res./Rev. Mutat. Res..

[33] Ferreira S.M., Lerner S.F., Brunzini R., Evelson P.A., Llesuy S.F. (2004). Oxidative stress markers in aqueous humor of glaucoma patients. Am. J. Ophthalmol..

[34] Goyal A., Srivastava A., Sihota R., Kaur J. (2014). Evaluation of oxidative stress markers in aqueous humor of primary open angle glaucoma and primary angle closure glaucoma patients. Curr. Eye Res..

[35] Nakajima Y., Inokuchi Y., Nishi M., Shimazawa M., Otsubo K., Hara H. (2008). Coenzyme Q10 protects retinal cells against oxidative stress in vitro and in vivo. Brain Res..

[36] Nakajima Y., Inokuchi Y., Shimazawa M., Otsubo K., Ishibashi T., Hara H. (2008). Astaxanthin, a dietary carotenoid, protects retinal cells against oxidative stress in-vitro and in mice in-vivo. J. Pharm. Pharmacol..

[37] Nakajima Y., Shimazawa M., Otsubo K., Ishibashi T., Hara H. (2009). Zeaxanthin, a retinal carotenoid, protects retinal cells against oxidative stress. Curr. Eye Res..

[38] Shimazawa M., Nakajima Y., Mashima Y., Hara H. (2009). Docosahexaenoic acid (DHA) has neuroprotective effects against oxidative stress in retinal ganglion cells. Brain Res..

[39] Lambuk L., Jafri A.J.A., Iezhitsa I., Agarwal R., Bakar N.S., Agarwal P., Abdullah A., Ismail N.M. (2019). Dose-dependent effects of NMDA on retinal and optic nerve morphology in rats. Int. J. Ophthalmol..

[40] Almasieh M., Wilson A.M., Morquette B., Cueva Vargas J.L., Di Polo A. (2012). The molecular basis of retinal ganglion cell death in glaucoma. Prog. Retin. Eye Res..

[41] Kaarniranta K., Koskela A., Felszeghy S., Kivinen N., Salminen A., Kauppinen A. (2019). Fatty acids and oxidized lipoproteins contribute to autophagy and innate immunity responses upon the degeneration of retinal pigment epithelium and development of age-related macular degeneration. Biochimie.

[42] Shukla U.V., Tripathy K. (2022). Diabetic Retinopathy. StatPearls.

[43] Brownlee M. (2005). The pathobiology of diabetic complications: A unifying mechanism. Diabetes.

[44] Du X., Matsumura T., Edelstein D., Rossetti L., Zsengellér Z., Szabó C., Brownlee M. (2003). Inhibition of GAPDH activity by poly(ADP-ribose) polymerase activates three major pathways of hyperglycemic damage in endothelial cells. J. Clin. Investig..

[45] Wu Y., Tang L., Chen B. (2014). Oxidative stress: Implications for the development of diabetic retinopathy and antioxidant therapeutic perspectives. Oxidative Med. Cell. Longev..

[46] Kuroki M., Voest E.E., Amano S., Beerepoot L.V., Takashima S., Tolentino M., Kim R.Y., Rohan R.M., Colby K.A., Yeo K.T. (1996). Reactive oxygen intermediates increase vascular endothelial growth factor expression in vitro and in vivo. J. Clin. Investig..

[47] Feldman E.L. (2003). Oxidative stress and diabetic neuropathy: A new understanding of an old problem. J. Clin. Investig..

[48] Garcia-Medina J.J., Pinazo-Duran M.D., Garcia-Medina M., Zanon-Moreno V., Pons-Vazquez S. (2011). A 5-year follow-up of antioxidant supplementation in type 2 diabetic retinopathy. Eur. J. Ophthalmol..

[49] Kowluru R.A., Kanwar M., Chan P.S., Zhang J.P. (2008). Inhibition of retinopathy and retinal metabolic abnormalities in diabetic rats with AREDS-based micronutrients. Arch. Ophthalmol..

[50] Shi C., Wang P., Airen S., Brown C., Liu Z., Townsend J.H., Wang J., Jiang H. (2020). Nutritional and medical food therapies for diabetic retinopathy. Eye Vis..

[51] Fleckenstein M., Keenan T.D.L., Guymer R.H., Chakravarthy U., Schmitz-Valckenberg S., Klaver C.C., Wong W.T., Chew E.Y. (2021). Age-related macular degeneration. Nat. Rev. Dis. Primers.

[52] Corbo J.C. (2021). Vitamin A1/A2 chromophore exchange: Its role in spectral tuning and visual plasticity. Dev. Biol..

[53] Hussain R.M., Gregori N.Z., Ciulla T.A., Lam B.L. (2018). Pharmacotherapy of retinal disease with visual cycle modulators. Expert Opin. Pharmacother..

[54] Fang Y., Taubitz T., Tschulakow A.V., Heiduschka P., Szewczyk G., Burnet M., Peters T., Biesemeier A., Sarna T., Schraermeyer U. (2022). Removal of RPE lipofuscin results in rescue from retinal degeneration in a mouse model of advanced Stargardt disease: Role of reactive oxygen species. Free Radic. Biol. Med..

[55] Yang S., Zhou J., Li D. (2021). Functions and Diseases of the Retinal Pigment Epithelium. Front. Pharmacol..

[56] Hong Y., Liang Y.-P., Chen W.-Q., You L.-X., Ni Q.-F., Gao X.-Y., Lin X.-R. (2021). Protective effects of upregulated HO-1 gene against the apoptosis of human retinal pigment epithelial cells in vitro. Int. J. Ophthalmol..

[57] The Age-Related Eye Disease Study Research Group (1999). The Age-Related Eye Disease Study (AREDS): Design implications. AREDS report no. 1. Control. Clin. Trials.

[58] Chew E.Y., Clemons T., SanGiovanni J.P., Danis R., Domalpally A., McBee W., Sperduto R., Ferris F.L. (2012). The Age-Related Eye Disease Study 2 (AREDS2): Study design and baseline characteristics (AREDS2 report number 1). Ophthalmology.

[59] Campochiaro P.A., Mir T.A. (2018). The mechanism of cone cell death in Retinitis Pigmentosa. Prog. Retin. Eye Res..

[60] Shen J., Yang X., Dong A., Petters R.M., Peng Y.-W., Wong F., Campochiaro P.A. (2005). Oxidative damage is a potential cause of cone cell death in retinitis pigmentosa. J. Cell. Physiol..

[61] Usui S., Komeima K., Lee S.Y., Jo Y.-J., Ueno S., Rogers B.S., Wu Z., Shen J., Lu L., Oveson B.C. (2009). Increased Expression of Catalase and Superoxide Dismutase 2 Reduces Cone Cell Death in Retinitis Pigmentosa. Mol. Ther..

[62] Abraham J., Lewis G. (2000). Regulating Medicines in Europe: Competition, Expertise and Public Health.

[63] Kessler D.A., Hass A.E., Feiden K.L., Lumpkin M., Temple R. (1996). Approval of new drugs in the United States. Comparison with the United Kingdom, Germany, and Japan. Jama.

[64] Ogrodnik P. (2019). Medical Device Design: Innovation from Concept to Market.

[65] Castellanos Rey L.C., Villamil Jiménez L.C., Romero Prada J.R. (2004). Incorporation of the Hazard Analysis and Critical Control Point system (HACCP) in food legislation. Rev. De Salud Publica.

[66] Kocabora M.S., Ozbilen K.T., Altunsoy M., Ahishali B., Taskapili M. (2008). Clinicopathological features of ocular cystinosis. Clin. Exp. Ophthalmol..

[67] Makuloluwa A.K., Shams F. (2018). Cysteamine hydrochloride eye drop solution for the treatment of corneal cystine crystal deposits in patients with cystinosis: An evidence-based review. Clin. Ophthalmol..

[68] Moura F.A., de Andrade K.Q., dos Santos J.C., Goulart M.O. (2015). Lipoic Acid: Its antioxidant and anti-inflammatory role and clinical applications. Curr. Top. Med. Chem..

[69] Ang M.J., Afshari N.A. (2021). Cataract and systemic disease: A review. Clin. Exp. Ophthalmol..

[70] Forman H.J., Zhang H., Rinna A. (2009). Glutathione: Overview of its protective roles, measurement, and biosynthesis. Mol. Asp. Med..

[71] Kang Q., Yang C. (2020). Oxidative stress and diabetic retinopathy: Molecular mechanisms, pathogenetic role and therapeutic implications. Redox Biol..

[72] Wang J., Li M., Geng Z., Khattak S., Ji X., Wu D., Dang Y. (2022). Role of Oxidative Stress in Retinal Disease and the Early Intervention Strategies: A Review. Oxidative Med. Cell. Longev..

[73] Young A.J., Lowe G.M. (2001). Antioxidant and prooxidant properties of carotenoids. Arch. Biochem. Biophys..

[74] Pescina S., Carra F., Padula C., Santi P., Nicoli S. (2016). Effect of pH and penetration enhancers on cysteamine stability and trans-corneal transport. Eur. J. Pharm. Biopharm..

[75] Saari J.C. (2012). Vitamin A metabolism in rod and cone visual cycles. Annu. Rev. Nutr..

[76] Blaner W.S., Shmarakov I.O., Traber M.G. (2021). Vitamin A and Vitamin E: Will the Real Antioxidant Please Stand Up?. Annu. Rev. Nutr..

[77] Scholl H.P., Moore A.T., Koenekoop R.K., Wen Y., Fishman G.A., van den Born L.I., Bittner A., Bowles K., Fletcher E.C., Collison F.T. (2015). Safety and Proof-of-Concept Study of Oral QLT091001 in Retinitis Pigmentosa Due to Inherited Deficiencies of Retinal Pigment Epithelial 65 Protein (RPE65) or Lecithin:Retinol Acyltransferase (LRAT). PLoS ONE.

[78] Maeda T., Cideciyan A.V., Maeda A., Golczak M., Aleman T.S., Jacobson S.G., Palczewski K. (2009). Loss of cone photoreceptors caused by chromophore depletion is partially prevented by the artificial chromophore pro-drug, 9-cis-retinyl acetate. Hum. Mol. Genet..

[79] Koenekoop R.K., Sui R., Sallum J., van den Born L.I., Ajlan R., Khan A., den Hollander A.I., Cremers F.P.M., Mendola J.D., Bittner A.K. (2014). Oral 9-cis retinoid for childhood blindness due to Leber congenital amaurosis caused by RPE65 or LRAT mutations: An open-label phase 1b trial. Lancet.

[80] Korenfeld M.S., Robertson S.M., Stein J.M., Evans D.G., Rauchman S.H., Sall K.N., Venkataraman S., Chen B.L., Wuttke M., Burns W. (2021). Topical lipoic acid choline ester eye drop for improvement of near visual acuity in subjects with presbyopia: A safety and preliminary efficacy trial. Eye.

[81] Grzybowski A., Markeviciute A., Zemaitiene R. (2020). A Review of Pharmacological Presbyopia Treatment. Asia-Pac. J. Ophthalmol..

[82] Leung H.H., Galano J.M., Crauste C., Durand T., Lee J.C. (2020). Combination of Lutein and Zeaxanthin, and DHA Regulated Polyunsaturated Fatty Acid Oxidation in H_2_O_2_-Stressed Retinal Cells. Neurochem. Res..

[83] Clementi M.E., Lazzarino G., Sampaolese B., Brancato A., Tringali G. (2019). DHA protects PC12 cells against oxidative stress and apoptotic signals through the activation of the NFE2L2/HO-1 axis. Int. J. Mol. Med..

[84] Ruiz-Pastor M.J., Kutsyr O., Lax P., Cuenca N. (2021). Decrease in DHA and other fatty acids correlates with photoreceptor degeneration in retinitis pigmentosa. Exp. Eye Res..

[85] Powell N., Chaudhary S., Zaidi A. (2021). It Is Time for an Oil Change: Polyunsaturated Fatty Acids and Human Health. Mo. Med..

[86] Hughbanks-Wheaton D.K., Birch D.G., Fish G.E., Spencer R., Pearson N.S., Takacs A., Hoffman D.R. (2014). Safety assessment of docosahexaenoic acid in X-linked retinitis pigmentosa: The 4-year DHAX trial. Investig. Ophthalmol. Vis. Sci..

[87] Hoffman D.R., Hughbanks-Wheaton D.K., Pearson N.S., Fish G.E., Spencer R., Takacs A., Klein M., Locke K.G., Birch D.G. (2014). Four-year placebo-controlled trial of docosahexaenoic acid in X-linked retinitis pigmentosa (DHAX trial): A randomized clinical trial. JAMA Ophthalmol.

[88] Hoffman D.R., Hughbanks-Wheaton D.K., Spencer R., Fish G.E., Pearson N.S., Wang Y.-Z., Klein M., Takacs A., Locke K.G., Birch D.G. (2015). Docosahexaenoic Acid Slows Visual Field Progression in X-Linked Retinitis Pigmentosa: Ancillary Outcomes of the DHAX Trial. Investig. Ophthalmol. Vis. Sci..

[89] Wubben T.J., Talwar N., Blachley T.S., Gardner T.W., Johnson M.W., Lee P.P., Stein J.D. (2016). Rates of Vitrectomy among Enrollees in a United States Managed Care Network, 2001–2012. Ophthalmology.

[90] Teixeira A., Novais E.A., Badaró E., Lima A., Farah M.E., Belfort R. (2019). Experimental model to evaluate the benefits of lutein to prevent retinal phototoxicity during pars plana vitrectomy surgery using xenon source light illumination in rabbits. Int. J. Retin. Vitr..

[91] van den Biesen P.R., Berenschot T., Verdaasdonk R.M., van Weelden H., van Norren D. (2000). Endoillumination during vitrectomy and phototoxicity thresholds. Br. J. Ophthalmol..

[92] Ankamah E., Sebag J., Ng E., Nolan J.M. (2019). Vitreous Antioxidants, Degeneration, and Vitreo-Retinopathy: Exploring the Links. Antioxid..

[93] Pietras-Baczewska A., Nowomiejska K., Sztanke M., Toro M., Rejdak R. (2020). Antioxidants in the retina and vitreous—Current state of knowledge. Ophthalmol. J..

[94] Siegfried C.J., Shui Y.B. (2019). Intraocular Oxygen and Antioxidant Status: New Insights on the Effect of Vitrectomy and Glaucoma Pathogenesis. Am. J. Ophthalmol..

[95] Dikstein S., Maurice D.M. (1972). The metabolic basis to the fluid pump in the cornea. J. Physiol..

[96] Benson W.E., Diamond J.G., Tasman W. (1981). Intraocular irrigating solutions for pars plana vitrectomy. A prospective, randomized, double-blind study. Arch. Ophthalmol..

[97] Rosenfeld S.I., Waltman S.R., Olk R.J., Gordon M. (1986). Comparison of intraocular irrigating solutions in pars plana vitrectomy. Ophthalmology.

[98] Nuyts R.M.M.A., Edelhauser H.F., Holley G.P. (1995). Intraocular irrigating solutions: A comparison of Hartmann’s lactated Ringer’s solution, BSS and BSS Plus. Graefe’s Arch. Clin. Exp. Ophthalmol..

[99] Cameron M.D., Poyer J.F., Aust S.D. (2001). Identification of free radicals produced during phacoemulsification. J. Cataract Refract. Surg..

[100] Ahmed S.S., Lott M.N., Marcus D.M. (2005). The Macular Xanthophylls. Surv. Ophthalmol..

[101] Stahl W., Sies H. (2003). Antioxidant activity of carotenoids. Mol. Asp. Med..

[102] Maia M., Furlani B.A., Souza-Lima A.A., Martins D.S., Navarro R.M., Belfort R.J. (2014). LUTEIN: A New Dye for Chromovitrectomy. Retina.

[103] Badaro E., Furlani B., Prazeres J., Maia M., Lima A.A., Souza-Martins D., Muccioli C., Lucatto L.F., Belfort R. (2014). Soluble lutein in combination with brilliant blue as a new dye for chromovitrectomy. Graefe’s Arch. Clin. Exp. Ophthalmol..

[104] Romano M.R., Ilardi G., Ferrara M., Cennamo G., Parolini B., Mariotti C., Staibano S., Cennamo G. (2018). Macular peeling-induced retinal damage: Clinical and histopathological evaluation after using different dyes. Graefe’s Arch. Clin. Exp. Ophthalmol..

[105] Olfat N., Ashoori M., Saedisomeolia A. (2022). Riboflavin is an antioxidant: A review update. Br. J. Nutr..

[106] Seiler T.G., Batista A., Frueh B.E., Koenig K. (2019). Riboflavin Concentrations at the Endothelium During Corneal Cross-Linking in Humans. Investig. Ophthalmol. Vis. Sci..

[107] Lombardo G., Serrao S., Lombardo M. (2020). Comparison between standard and transepithelial corneal crosslinking using a theranostic UV-A device. Graefe’s Arch. Clin. Exp. Ophthalmol..

